# Arthroscopic Latarjet for Recurrent Shoulder Instability

**DOI:** 10.3390/medicina55090582

**Published:** 2019-09-11

**Authors:** Roberto Castricini, Umile Giuseppe Longo, Stefano Petrillo, Vincenzo Candela, Massimo De Benedetto, Nicola Maffulli, Vincenzo Denaro

**Affiliations:** 1 Department of Orthopaedic and Trauma Surgery, Villa Maria Cecilia Hospital, 48033 Cotignola, Ravenna, Italy; r.castricinicbm@gmail.com (R.C.); debenedetto@campusmail.com (M.D.B.); 2 Department of Orthopaedic and Trauma Surgery, Campus Bio-Medico University, 00128 Rome, Italy; petrillocbm@gmail.com (S.P.); v.candela@unicampus.it (V.C.); denaroucbm@gmail.com (V.D.); 3 Centre for Sports and Exercise Medicine, Barts and The London School of Medicine and Dentistry, Mile End Hospital, Bancroft Rd, Bethnal Green, London E1 4DG, UK; maffulliucbm@gmail.com

**Keywords:** arthroscopy, shoulder instability, trauma, arthroscopic Latarjet, anterior recurrent instability

## Abstract

*Background and Objectives:* The all-arthroscopic Latarjet (aL) procedure was introduced to manage recurrent shoulder instability. Our study aimed to report the outcomes of aL procedures with the Rowe, University of California-Los Angeles (UCLA), simple shoulder test (SST) scores, and range of motion (ROM) in external rotation at a minimum follow-up of 2 years. *Material and Methods:* A total of 44 patients presenting recurrent shoulder instability were managed with aL procedure. Clinical outcomes were assessed at a mean follow-up of 29.6 ± 6.9 months. The postoperative active ROM was measured and compared with the contralateral shoulder. The Rowe, UCLA, and SST scores were administered preoperatively and postoperatively. *Results:* No patients experienced infections or neuro-vascular injuries. Seven (15%) patients required revision surgery. After surgery, the external rotation was statistically lower compared to the contralateral shoulder, but it improved; clinical outcomes also improved in a statistically significant fashion. *Conclusions:* The aL produced good results in the management of recurrent shoulder instability, but the complication rate was still high even in the hands of expert arthroscopist.

## 1. Introduction

The management of traumatic anterior shoulder instability represents a challenge, especially in young and active patients [1]. Arthroscopic Bankart repair provides good early results, but approximately 50% of failures occur more than 2 years after the initial surgery [2], and the procedure is not as successful as open capsulolabral repair [1,3,4,5]. Moreover, in patients with the marked bone loss associated with a capsular deficiency from chronic instability [6,7,8], Bankart repair alone does not prevent recurrent instability, and different bone-block techniques have been developed in the last few years [3].

In the Latarjet procedure, an osteotomy of the horizontal part of the coracoid process at its elbow is performed, and the coracoid process is then transferred and internally fixed to the glenoid rim. The coracoid graft is positioned subequatorially in the vertical plane, and flush to the glenoid surface in the horizontal plane, and it is fixed to the glenoid rim with two bicortical screws [9,10]. This procedure can produce excellent results, with a low rate of recurrent instability and high rate of return to sports at pre-injury level [11,12,13].

From a biomechanical viewpoint, the Latarjet procedure increases the anteroposterior diameter of the glenoid and reinforces the anterior capsule with the coracoacromial ligament. Moreover, the coracoid transfer to the glenoid rim produces a sling effect, also called “hammock effect”, produced on the humeral head by the conjoined tendon, avoiding shoulder dislocation when the arm is in the abduction and external rotation [14].

Following the evolution of arthroscopic techniques, several authors described the all-arthroscopic Latarjet (aL) procedure. Arthroscopy may allow good positioning of the coracoid process [15,16,17,18,19] because imaging is magnified. Moreover, it allows managing associated lesions, such as cuff tears and labrum lesions [20,21].

The present study aimed to report the effectiveness of the aL procedure with the Rowe score, University of California-Los Angeles (UCLA) score, simple shoulder test (SST) score, and range of motion (ROM) in external rotation at a minimum follow-up of 2 years.

## 2. Materials and Methods

The present study was approved by the local Ethics committee of Campus Bio-Medico of Rome. The Ethical code approval number was 37/16 OSS. The date of approval was 14 September 2016. All participants were recruited at the University Hospital Campus Bio-Medico of Rome and gave written consent to participate.

### 2.1. Patient Selection

From March 2013 to June 2014, 54 patients presenting recurrent shoulder instability were managed with the aL procedure. Patients who had undergone previous surgery for shoulder instability were also included. Patients were excluded from the study if at the time of surgery they presented—inflammatory joint disease, degenerative arthritis of the glenohumeral joint, symptomatic arthritis of the acromioclavicular joint, or cuff tear arthropathy.

Preoperative and postoperative clinical and imaging data were collected prospectively. All patients were managed with the same aL procedure and postoperative rehabilitation. Follow-up evaluation at a minimum of two years included: questionnaires for the evaluation of the shoulder function; physical examination; anteroposterior and lateral radiographs; computer tomography (CT) scans to evaluate graft positioning and healing, and screws position.

### 2.2. Clinical Assessment

A surgeon not involved in the surgery performed a standardized postoperative physical examination of all patients at a mean follow-up of 29.6 ± 6.9 months (range 24–72 months). The range of motion (ROM) was measured with a goniometer, according to standard measurements guidelines. The active ROM in the external rotation was measured with the arm adducted and the elbow flexed at 90 degrees (ER1), and with the arm abducted at 90 degrees and the elbow flexed at 90 degrees (ER2). The value obtained in the operated shoulder was compared with the contralateral. Three measurements were recorded for each ROM considered, and the mathematical average was used for statistical purposes [22].

The Rowe score [23] was used to evaluate preoperative and postoperative shoulder motion, function, and stability. The maximum score obtainable is 100 points. The lower the score, the worst the condition of the shoulder. The modified University of California, Los Angeles (UCLA) [23] shoulder rating scale was used to evaluate preoperative and postoperative shoulder pain, function, active forward flexion, strength, and patients’ satisfaction. The maximum score obtainable is 35 points, and the results were classified as excellent (34–35 points), good (28–33), fair (21–27), or poor (0–20).

The Simple shoulder test (SST) [23] was used to evaluate preoperative and postoperative shoulder function and the influence of shoulder disability on activities of daily living. The maximum score obtainable is 12 points. The lower the score, the worst the condition of the shoulder.

### 2.3. Imaging Assessment

Imaging assessment was performed in all patients by a musculoskeletal radiologist with a special interest in shoulder pathology.

**Figure 1:**
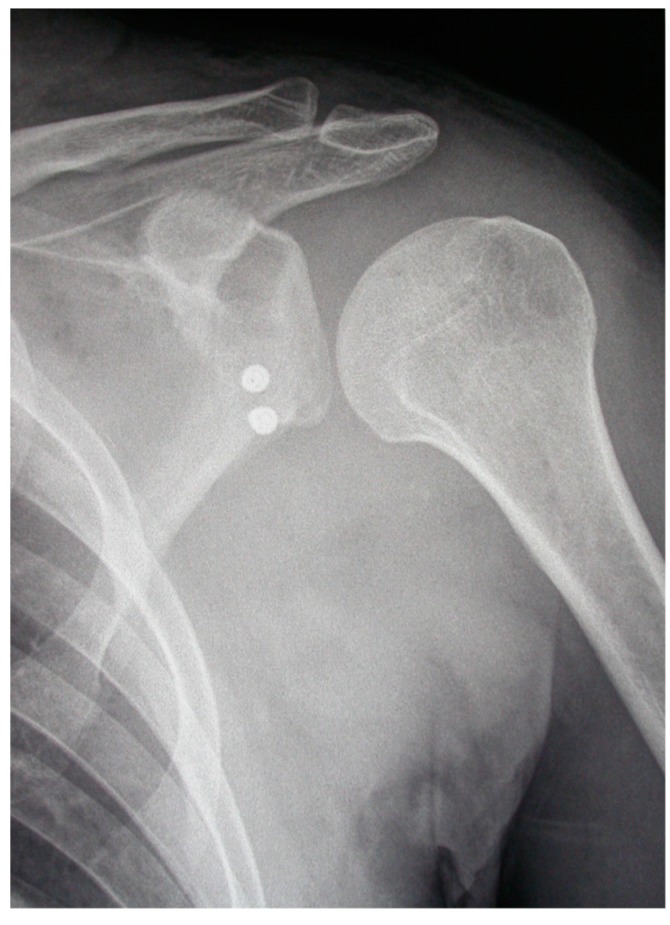
Postoperative X-ray control showing that the graft has perfect positioning.

Postoperatively, radiographic assessment of the coracoid graft positioning in a true anteroposterior and lateral view was performed 24 h and one month after surgery in all patients (Figure 1).

CT scans were performed in 34 (77%) patients to assess graft positioning, healing, and the angle between the screws and the glenoid surface at a mean follow-up of 14 ± 8.8 months (range 6–36 months) after surgery. In the remaining 10 (23%) patients, CT scans were not performed. Coracoid graft positioning in the axial plane was classified, according to Boileau [15] classification, as flush to the glenoid surface (correct graft position), too medial (>5 mm medial to glenoid rim), and too lateral (>5 mm lateral to glenoid rim). Graft positioning in the coronal plane was assessed according to Lafosse description [24].

### 2.4. Surgical Technique

**Figure 2:**
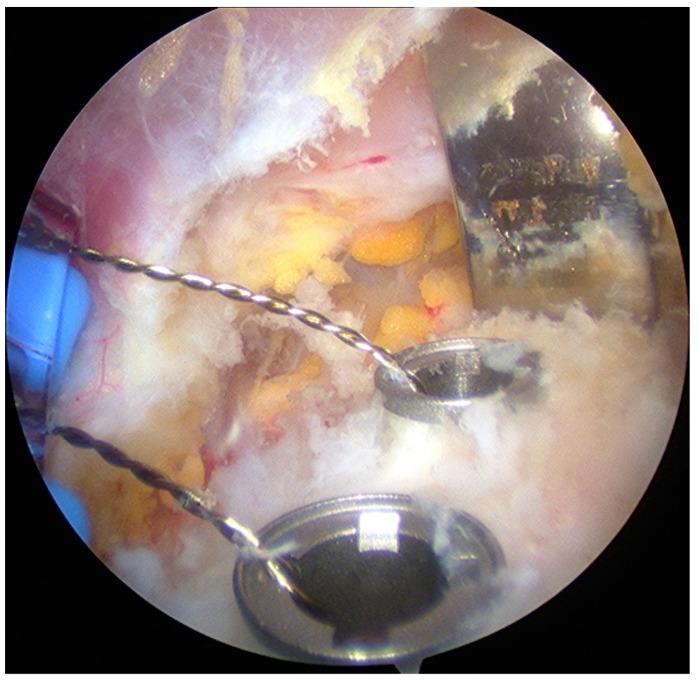
Coracoid osteotomy.

**Figure 3:**
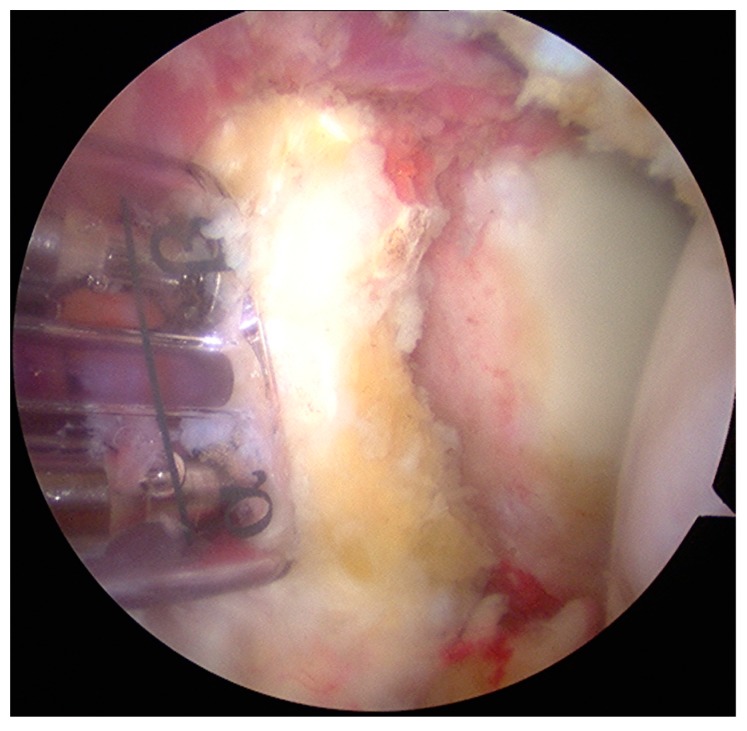
The graft is mobilized through the subscapularis split, using the double cannula, on the anterior glenoid neck.

**Figure 4:**
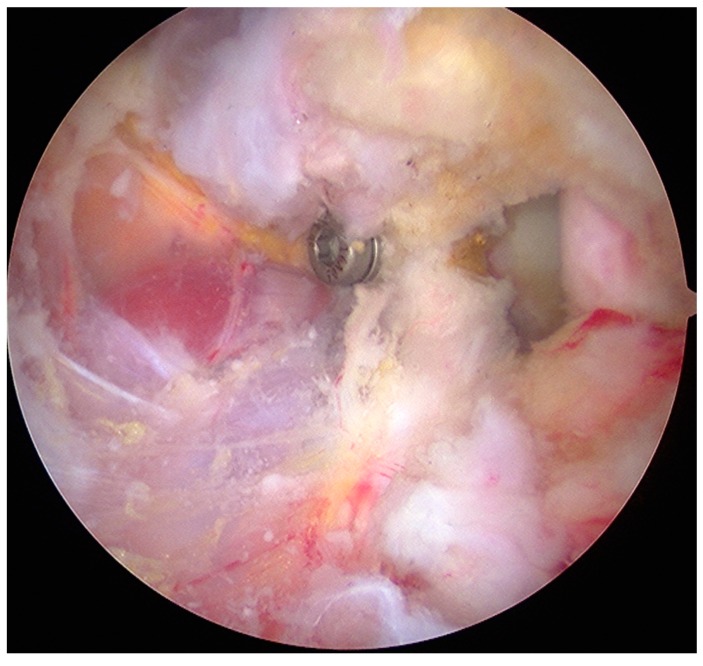
Final view from anteroinferior portal. Subscapularis tendon covers the proximal screw.

The same surgeon performed all aL procedures. The surgical procedure could be divided into 5 stages [25]:

1. Joint evaluation and exposure through a posterior portal and an anterior portal. The lateral side of the coracoids was analyzed, opening the rotator interval. The coracoacromial ligament was released.
2. Subscapularis split through the anterolateral portal (parallel to the superior margin of the subscapularis tendon). In this stage, an inferior portal at the apex of the anterior axillary fold, an anteroinferior portal midway between the inferior and the anterolateral portal, and a medial portal were performed. The split area was identified through the posterior portal; the conjoint tendon and the brachial plexus were protected through the medial portal with a switching stick. A burr was used to expose and abrade the anterior wall of the glenoid neck.
3. The pectoralis minor was released, and a fresh portal was established with a needle over the coracoid, to insert the drill holes, at the junction of the lateral 2/3 and the medial 1/3. Two Kirschner wires through the coracoid were passed. The drill guide was removed. The holes were tapped, and the top hats were inserted into the fragment using a flexible Chia wire. The osteotomy was completed (Figure 2). The bone fragment was secured thought a coracoid screw passed over the Chia wire, using a double cannula.
4. The graft was positioned on the anterior glenoid neck with the switching stick through the posterior portal (Figure 3).
5. The graft was fixed. Two holes were made through the coracoid and the glenoid using the double cannula. Graft trimming was performed with the burr (Figure 4).

Two 3.5 mm cannulated bicortical screws were placed to fix the graft to the glenoid rim in all patients, except for one patient, in whom only one screw was used. The mean duration of the surgical procedure was 105 ± 28 min (range 70–230 min).

### 2.5. Postoperative Rehabilitation

A sling with a pillow in slight internal rotation and 10° of abduction was used for 4 weeks. Passive external rotation exercises to 0° (straight-ahead position) were allowed immediately. Overhead stretches and pulley were allowed after 4 weeks. External rotation stretching was progressed to 50% of the external rotation of the contralateral shoulder by 12 weeks. Internal rotation stretching was commenced to regain 80% of the internal rotation of the contralateral shoulder by 12 weeks. After 6 weeks, isoinertial concentric strengthening and rehabilitation of the rotator cuff, deltoid, and scapular stabilizers were allowed. The mean rehabilitation period was 3 months (range 1–10 months). Heavy manual work was allowed at a mean of 4 months (range 2–6 months) after surgery, while light manual work started at a mean of 1.2 months (range 1–3 months) after surgery. Return to the sport was allowed at a mean of 5.5 months (range 1–12 months).

### 2.6. Data Collection and Statistical Analysis

Data were collected in a computer database and included demographic information, physical examination findings, outcome scores results, and imaging results. 

The statistical significance of improvement of the outcome parameters was assessed using the paired Wilcoxon signed-rank test. *p*-values lower than 0.05 were considered statistically significant.

## 3. Results

### 3.1. Demographics

The study group included 44 patients (44 shoulders). There were 42 (95%) (42/44) male and two (5%) (2/44) female patients, with a mean age of 29.8 ± 8.9 years (range 18–51 years). The right shoulder was involved in 19 (43%) (19/44) patients, and the left shoulder was in 25 (57%) (25/44) patients. Seven (16%) (7/44) patients had undergone prior surgery: six (14%) (6/44) patients had arthroscopic Bankart repair and one (2%) (1/44) patient underwent open Putti–Platt procedure. No patients experienced infections or neuro-vascular injuries. Five (11.3%) (5/44) patients had a failure. Other seven (18%) (7/44) patients developed complications. Six (16%) (6/44) of these underwent re-operation (Table 1).

### 3.2. Clinical Outcomes

The clinical evaluation was performed in 39 patients at a mean follow up of 29.6 ± 6.9 months (range 24–72 months) after surgery. The Rowe score, UCLA score, and SST were administered preoperatively and at the last follow-up evaluation. Postoperatively, the ER1 averaged 67.7 ± 12.3 degrees (range 30–85 degrees) in the operated shoulder and 75.5 ± 6.7 degrees (range 60–85 degrees) in the contralateral shoulder (*p* = 0.001). The ER2 averaged 81.5 ± 9.3 degrees (range 60–95 degrees) in the operated shoulder and 86.5 ± 4.9 degrees (range 80–95 degrees) in the contralateral shoulder (*p* = 0.01).

The average preoperative Rowe score was 27.2 ± 4.6 points (range 15–50 points), and the average postoperative Rowe score was 90.2 ± 11.1 points (range 50–100) (*p* < 0.0001).

The average preoperative UCLA shoulder score was 21.6 ± 1.8 points (range 18–24), and the average postoperative UCLA shoulder score was 32.5 ± 2 points (range 27–35) (*p* < 0.0001)*. After surgery, according to the UCLA shoulder rating system, in 12 patients (27%), the results were considered excellent, in 31 patients (71%) good, and in one patient (2%) fair.

The average preoperative SST was 8.9 ± 0.9 points (range 7–10 points), and the average postoperative SST was 11.6 ± 0.6 points (range 9–12 points) (*p* < 0.0001). Thirty-three (75%) patients were very satisfied, nine (21%) patients were satisfied, one (2%) patient was quite satisfied, and one (2%) patient was not satisfied after surgery.

### 3.3. Imaging Outcomes

#### 3.3.1. Coracoid Graft Healing

Coracoid graft healing was assessed by CT scanning in 34 (87%) (34/39) patients at a mean follow-up of 14 ± 8.8 months (range 6–36 months). Thirty (88%) (30/34) patients experienced complete healing of the graft. Of these, four (14%) (4/34) patients developed postoperative lysis. The remaining four (12%) (4/34) patients presented a nonunion/fibrous union of the graft.

#### 3.3.2. Coracoid Graft Positioning

**Figure 5:**
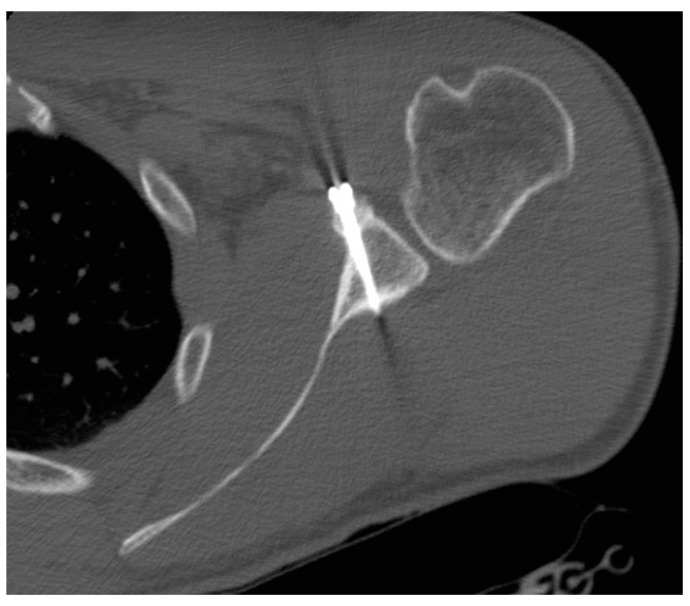
Axial computed tomography scan of a united coracoid graft.

**Figure 6:**
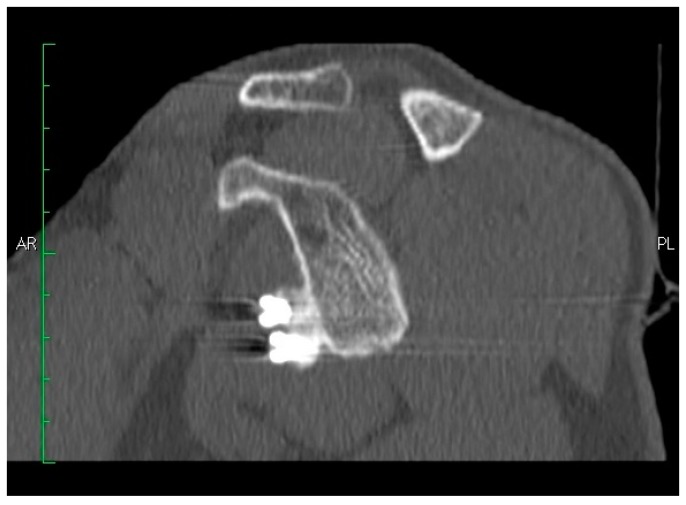
Sagittal computed tomography scan of a united coracoid graft.

Coracoid graft positioning was assessed in 34 (87%) (34/39) patients with CT scans and five (13%) (5/39) patients with radiographs at a mean follow-up of 14 ± 8.8 months (range 6–36 months) and one month after surgery, respectively. In the coronal plane, in 38 (86%) (38/44) patients, the coracoid graft was in the optimal subequatorial position; in three (7%) (3/44) patients, it was lower than the optimal position, and in three (7%) (3/44) patients, the coracoid graft was higher than the optimal position. In the axial plane, the coracoid graft was in the optimal position, i.e., flush with the glenoid surface, in 41 (93%) (41/44) patients; in 3 (7%) (3/44) patients, the coracoid graft was too medial to the glenoid surface (Figure 5 and Figure 6).

#### 3.3.3. Screws Angle

The angle between the screws and the glenoid surface was assessed with CT scanning in 34 patients at a mean follow-up of 14 ± 8.8 months (range 6–36 months): it was 28.3 ± 8 degrees (range 12–45 degrees). No screw migration occurred, and no screw penetrated the joint (Figure 5 and Figure 6).

## 4. Discussion

The present study evaluated the effectiveness of aL procedure for the management of patients with recurrent anterior shoulder instability through clinical assessment and imaging results. Almost 30 months after aL procedure, the Rowe, UCLA, and SST scores significantly improved, and only one patient was not satisfied after surgery. However, 11.3% of patients had a failure, and the operated shoulder had lower external rotation ROM (ER1 and ER2) when compared with the contralateral shoulder.

Arthroscopy allowed a safe identification of the axillary nerve and vascular structures [26] capable of avoiding nerve or vascular or joint injuries. However, the coracoid graft fractured in three (7%) instances: two were detected with radiographs the day after the index surgery, and the latter occurred 20 days after the index surgery. Reoperation was necessary for seven (15%) patients because of (1) post-traumatic posterior shoulder dislocation; (2) screws intolerance; (3) recurrent instability, and (4) coracoid graft fracture. This complication rate was higher than what reported in a recent systematic review, which showed a 1.5% rate of intra- or postoperative fracture, 3.2% of postoperative lysis, and 9.4% of nonunion/fibrous union of the graft [27,28].

In our series, the graft had healed in 88% (30/34) of patients, but 14% (4/34) of these had postoperative lysis. In the coronal plane, in 86% (38/44) of patients, the coracoid graft was in the optimal subequatorial position, while in the axial plane, 93% (41/44) of patients presented the coracoid graft in the optimal position, flush with the glenoid surface. Boileau et al. [15,17] reported that 89% of the coracoid grafts were in the optimal position (below the equator and flush to the glenoid surface), founding also 98% and 91% of optimal positioning of the coracoid graft in the axial plane and coronal plane, respectively. When considering the open Bristow–Latarjet procedure, Hovelius et al. [20] reported that, in a cohort of 85 patients, the coracoid graft was too high in 49% of cases, while Walch et al. [29] found that 27% of the transferred coracoid bone grafts were too lateral, and 12% too medial. Proper positioning of the bone block in both the axial and coronal planes is crucial to obtain excellent results. Proper positioning of the bone block in both the axial and coronal plane is crucial to obtain excellent results. If the coracoid bone block had been placed too medial or too high, recurrent instability was likely [29,30,31]. On the other hand, if the coracoid bone block had been placed too lateral in an overhanging position, late glenohumeral osteoarthritis was likely [29,30,31]. In open Bristow–Latarjet procedures, the malpositioning, of both bone block and screw, occurs in over 50% of patients [30,31,32]. 

In the present investigation, the coracoid graft was fixed to the glenoid rim with two bicortical screws in all but one patient. In our series, the mean angle between the screws and the glenoid surface was 28.3 ± 8 ° (range 12–45 °), in the agreement of the literature available [18,24]. Arthroscopy allows good positioning of both coracoid graft and screws. Furthermore, arthroscopy offers the possibility to manage the associated lesions of the shoulder, including rotator cuff tears, pathology of the long head of the biceps tendon, SLAP lesions, and lesions of the posterior labrum [3,16,17,18,24].

A major strength of the present study is that a single fully trained senior orthopedic surgeon performed all the aL procedures, while an orthopedic surgeon who was not involved in the index surgery performed all the clinical and functional evaluations. Moreover, the evaluation of ROM was performed according to standard measurements guidelines. Finally, we included patients with a minimum follow-up of 24 months. Another important strength of the study is that a radiologist performed the radiographic and CT scans analysis, assessing the coracoid graft healing and measuring the angle between the screws and the glenoid surface. 

Research in the setting of shoulder instability is growing, even though several unsolved problems remain [1,33,34,35,36,37,38]. The recent concept of the glenoid track is challenging our understanding of bone loss; even though, the application to the clinical practice of this concept is not always easy, especially outside of specialized centers. Research in this field is needed to allow shoulder surgeons to provide the best possible clinical outcome to patients. 

## 5. Conclusions

The arthroscopic Latarjet procedure can be considered a promising solution to manage recurrent anterior shoulder instability, but senior shoulder surgeons very confident with arthroscopic techniques should perform it. 

## Figures and Tables

**Table 1:** Complications.

Complications	Number of Patients (%)	Management
Coracoid graft rupture	3 (7%)	Surgery: screws removal
Screws intolerance	2 (5%)	Surgery: screws removal
Hematoma	1 (2%)	Conservative management
Recurrent instability	1 (2%)	Surgery (other Hospital)
